# Supplementary material for: Relationship of vitamin D, fibrinogen and their ratio with acute coronary syndrome: A comparative analysis of unstable angina, NSTEMI, and STEMI
Source: PLoS One. 2025 Sep 5;20(9):e0331853. doi: 10.1371/journal.pone.0331853 (PMC12412923; doi:10.1371/journal.pone.0331853)
Supplement: Attachment — Submitted filename: Response to Reviewers.docx [file pone.0331853.s003.docx]

Point By Point Response to Reviewers’ Comments:

| Reviewer Comments | Authors’ Response |
| --- | --- |
| Reviewer # 1  Overall, the association between vitamin D, fibrinogen, and their ratio with acute coronary syndrome (ACS), which is innovative and clinically significant, however there is still room for improvement in research methods, data presentation and discussion. While the study's use of non-probability convenience sampling achieved baseline feasibility, this methodological approach introduces inherent selection bias that may compromise the external validity of findings. It is recommended that subsequent studies adopt random sampling or other more scientific sampling methods to ensure the representativeness of the samples. The sample size of 300 subjects demonstrates borderline adequacy for investigating complex biomarker interactions in ACS pathogenesis, potentially resulting in diminished statistical power to detect clinically meaningful associations.. It is possible to consider expanding the sample size for more in-depth analysis. The selection of statistical methods is basically reasonable, but given the limitations of the sampling method, its impact on the statistical results should be further evaluated in the discussion of the results. At the same time, in-depth analysis of the clinical significance of the statistical results can be increased. Although the discussion part compares the research results with previous studies, The discussion would benefit from expanded consideration of translational applications for the vitamin D/fibrinogen ratio, particularly concrete recommendations for clinical implementation and biomarker-guided therapeutic strategies. The current conclusions are supported by certain data, but they need to be interpreted with caution given the limitations of the study. It is recommended to more clearly point out the impact of the limitations of the study on the generalizability of the conclusions in the conclusion. Some content is repeated, such as the repeated mention of the research background in the introduction and discussion. The content can be streamlined to make the discussion clearer. Certain complex sentence structures may impede readability and could be streamlined for improved clarity. While ethical approval was obtained, enhanced documentation of patient privacy safeguards would strengthen methodological transparency. Furthermore, future investigations should examine potential ethnic and geographic variations in biomarker relationships to enhance the generalizability of findings across diverse populations | Thanks for the valuable comments and input into the improvement of the manuscript and really appreciate your kind words regarding the study.  Regarding the sampling technique, we totally agree that random sampling is the ideal technique, but the non-probability convenience sampling was more practical choice due to the emergency nature of ACS and the access constraints in three different international centers, having different patient record management systems. However, we have added this to the limitations section in the revised manuscript.  Regarding the sample size of 300 patients, we agree that larger studies would increase statistical power and external validity, our sample well exceeded the minimum calculated threshold of 60 patients. Moreover, we acknowledge this and have added a note encouraging future larger-scale studies.  Regarding the effects of sampling on statistical results, we have included a brief discussion of how the non-probability design may have influenced the statistical estimates, and what cautions have been done for analysing the effect sizes and confidence intervals with marginal p values in the discussion of the results.  Regarding the translational application of the vitD/fibrinogen ratio, the discussion is further strengthened by clinical implication of the same in the revised manuscript as suggested. The revised manuscript now elaborates on how the ratio could be integrated into existing clinical workflows and risk scores in adjunct to troponins or CRP.  Regarding the Conclusion, the sentence is added as, “These findings, while novel, require cautious interpretation given methodological limitations and need further validation,” in order to give a more cautious statement based on the study limitations.  Regarding the repetition and redundancy in the introduction and discussion, the revised manuscript has removed several points like repeating introductory text from the Discussion describing the ACS subtypes.  The manuscript is revised with the help of professional copyediting and proofreading, making it simpler and easier to understand.  Regarding ethics and patient privacy, additional explanation has been added to Methodology section mentioning the anonymisation of the data and authorized access to the patient data.  Regarding the ethnic and geographic variations and generalizability, while we did stratify by country and found no statistically significant differences as mentioned Brown-Forsythe test , we have further emphasized its importance in the discussion. |
| Reviewer #2:  I have read this article in detail. This article mainly discusses the correlation between vitamin D/fibrinogen and different types of acute coronary syndrome (ACS). In the currently published articles, there is a lack of data on Arab patients in the Middle East, and this article effectively fills this gap. Unfortunately, there are still some issues that need to be addressed in this article.  1. Grammar and article structure I have to regretfully point out that the grammar and structure of this article are confusing. There is a significant difference in the quality of grammar in the article. In some parts, such as the beginning of the article, the language expression of the article has not reached the level of current mainstream articles. In addition, the production of tables and images is also quite rough. The position of the image is incomprehensible and placed at the end. The low resolution of the image makes it difficult to read.  2. References cited: The citation level of this article does not support the author's viewpoint. It is recommended to choose updated, more mainstream, and frequently cited articles when selecting references. And in terms of article type, it is recommended to choose more basic experimental or clinical papers rather than reviews. Additionally, there is a lack of references to the latest guidelines currently available.  3. Target selection: At the beginning of the article, the author did not provide a clear explanation for why the ratio of vitamin D to fibrinogen was chosen. This can cause confusion for readers. In the following content, the author claims that STEMI is more dangerous than NSTEMI. This seriously affects the conclusion of this article. In the 2017 ESC NSTEMI guidelines, it is recommended to use GRACE to evaluate the degree of danger. In this article, there is a lack of evaluation of NSTEMI, which makes the conclusion completely unsupported. From the logic of the article, the changes in vitamin D/fibrinogen are more like manifestations of ACS classification rather than the severity of ACS. | Thanks for your valuable input to the manuscript.  1. Regarding the Grammer and article structure we really appreciate your comments and the authors have revised the manuscript with the help of professional copyeditor and proofreader. The production of tables and figures are formatted according to the journal’s guidelines once article is accepted and sent for production. The figure will be placed where it is mentioned in the text, i.e., after table 4 and is prepared according to the journal guidelines having more than 300dpi. However, we have revised and improved the quality of the figure to 1200 dpi.  2. Regarding the cited references, 10 references are from the past 2021-2024, 7 from year 2010 to 2020, and 8 from 2001 to 2010, showing upto 65% of references are from the last 10 years and 40% from the last 3 years. None of the reference is older than 2001. Among these 12 are original clinical/observational studies, 3 metanalysis, 4 systematic reviews, 4 pathophysiologic and literature reviews and 2 are books chapters with guidelines, showing a healthy balance of the different components of scientific literature. These references are selected based on scientific relevance according to academic standards and not the citation count.  3. Regarding the comment, “At the beginning of the article, the author did not provide a clear explanation for why the ratio of vitamin D to fibrinogen was chosen”, I would humbly state that the first line of the abstract start with the statement, *“There is emerging evidence suggesting that vitamin D and fibrinogen play contrasting roles in ACS pathophysiology and their combined impact, expressed as the vitamin D/fibrinogen ratio, can be a potential biomarker for ACS severity.”* Similarly in the Introduction section, after the introduction of the ACS and the biomarkers in the question, the selection of their ratio is explained as, *“These contrasting effects of vitamin D and serum fibrinogen may give rise to the hypothesis that the interplay of these two important biomarkers could affect the course and severity of CAD.”* I hope this clarifies the rationale of using the vit D/fibrinogen ratio to the readers from the first line.  Regarding the comment, “*the author claims that STEMI is more dangerous than NSTEMI. This seriously affects the conclusion of this article”,* I would humbly state that it is not my claim, rather it is supported by published evidence as referenced in the manuscript and also supported by the pathophysiologic and vascular changes in STEMI as compared to NSTEMI (Table 1).  Regarding the comment, “*In the 2017 ESC NSTEMI guidelines, it is recommended to use GRACE to evaluate the degree of danger. In this article, there is a lack of evaluation of NSTEMI, which makes the conclusion completely unsupported.*” We agree that formal risk scores, such as GRACE or TIMI quantify the individual patient level severity, they do not capture the catogoric level severity of ACS sub-types. The GRACE score, which accounts for age, pulse rate, Systolic BP, creatinine, cardiac arrest, ST-segment deviation, abnormal cardiac enzymes and Killip class for the severity, is used for estimating the severity of individual ACS patients, and not the ACS sub-groups. However, even in the GRACE scoring system, the ST-segment deviation will always be positive for STEMI, but may be negative for NSTEMI, denoting the higher scores in STEMI Vs NSTEMI if the other components are equal. Having said that, none of the objectives of the study is related to the outcome of NSTEMI or any other type ACS; rather all the objectives are regarding the relation of vitamin D, fibrinogen and their ratio to the ACS sub-types. |
| Reviewer #3  **Major Comment:** While the study presents interesting statistical associations between vitamin D, fibrinogen, and ACS severity, it lacks discussion on potential mechanistic pathways or how these findings might translate into clinical decision-making. The authors should elaborate in the Discussion on the biological plausibility of the vitamin D/fibrinogen ratio affecting ACS pathophysiology and address how this biomarker could be incorporated into existing clinical workflows for risk stratification or therapeutic monitoring.  **Minor Comments:**  1. Overextended Introduction: The Introduction is overly long and includes detailed explanations that are more appropriate for the Discussion section, such as the rationale for comparing biomarkers and interpretation of their roles. The authors are encouraged to streamline the introduction to focus on background, knowledge gaps, and objectives, moving interpretive content to the Discussion.  2. Patient Selection Criteria Should Be Visualized: In the Methods section, the inclusion and exclusion criteria are clearly described in text, but a flowchart (e.g., a CONSORT-style diagram) would greatly enhance clarity and transparency, particularly in a multicenter study.  3. Clarify Country-Level Differences: The results mention no significant differences across countries, but no further stratified analysis or sensitivity analysis is provided. A brief comment on inter-country consistency or variability (e.g., healthcare systems, diagnostic protocols) would strengthen the generalizability claim.  4. Typographical and Formatting Issues Several instances of awkward phrasing (e.g., “To make things simple...”) and inconsistent formatting (e.g., line breaks, inconsistent p-value notation) are present throughout the manuscript. A thorough copyedit is recommended to improve readability and professionalism.  5. Clarify Ratio Interpretation: The interpretation of the vitamin D/fibrinogen ratio as a “stronger” correlate than vitamin D alone is not statistically supported (Fisher Z-test p = 0.73). This should be clearly stated in both Results and Conclusion to avoid overstating the findings. | Major Comment:  Thanks for highlighting the importance of addressing mechanistic plausibility and clinical application of our findings. We respectfully state that both of these points are elaborated in the discussion section as we state,  “*Their contrasting effect provided the rationale for this approach, since this ratio may prove as a potential biomarker for ACS types and severity… These findings strengthen the evidence in favour of our hypothesis, as clots in STEMI are the most stable clots as compared to unstable angina and NSTEMI.”* (Page ~17, Paragraph starting with “One of the novel aspects…)”. This paragraph provides the biological plausibility and mechanistic rationale to our hypothesis. In another paragraph in the “Clinical Implication” subsection of the Discussion, we state that,  “*The inverse relation of vitamin D and the direct relation of fibrinogen could serve as prognostic biomarkers of ACS severity. Secondly, these values could guide regarding the possible vitamin D supplementation and fibrinogen lowering therapies…”* (Page ~18, Paragraph starting with “The clinical implications of this study…”).  Moreover, in the revised manuscript we have added a paragraph stating,  *“In clinical practice, this may serve as a surrogate marker of severity and outcome, complementing the existing biomarkers such as troponin and CRP, particularly in patients where these traditional markers are equivocal.”*  Minor Comment:  1. Regarding the overextended Introduction, it has been revised, now having just 736 words (including a table), highlighting the importance of the study, gaps in the literature, rationale behind the study and the main objectives.  2. Regarding the CONSORT type flow diagram we appreciate the reviewer’s suggestion, however, we respectfully believe that in the context of our observational, cross-sectional study, such a diagram is not essential, as it is not a clinical trial, having no drop out of the patients and whole of the cohort is included in the study, and the inclusion and exclusion criteria with sample size is clearly and very simply stated. Adding a diagram may cause redundancy, however, if the editor deems it necessary for consistency or presentation purposes, we would be happy to include a simplified flow diagram in the final version.  3. Regarding the inter-county variability, the statistical analysis showed no significant differences in the variables among the ACS categories of the three counties as we state in the results, *“Brown-Forsythe test showed no significant difference among the patients from the three countries on the basis of vitamin D, fibrinogen and their ratio (P > .05 for all the three).”* Based on this fact, the patients of all the countries can be considered and analyzed as a single cohort and commenting on the variability may go against the statistical proof.  4. Regarding typographical and formatting issues, we acknowledge this issue and the revised manuscript is assessed through professional copy-editing and proofreading to improve this shortcoming.  5. Regarding the vit D/fibrinogrn ratio interpretation, we acknowledge the suggestion and have added a clear note to the conclusion that the ratio is not significantly stronger than isolated vitamin D levels statistically. |
| Reviewer #4:  1. Does Table 1 need to explain anything? Table 1 is just common clinical knowledge and doesn't seem to contribute to the paper.  2. What is the economic value of measuring vitamin D and fibrinogen in the assessment of acute myocardial infarction? What are the advantages over troponin and CK-MB? It seems to have little clinical significance.  3. The research has certain advantages in using data from multiple countries, but the sample size used in the study is too small, resulting in weak proof strength. | Thanks for the valuable input for improving the manuscript.  Regarding Table 1, we appreciate your comment, but we respectfully submit that Table 1 was included to simplify the differences of the ACS subtypes showing their severity to support the rationale for exploring the pro- and anti-thrombotic and pro- and anti-inflammatory biomarkers. This is to serve a pedagogical and contextual purpose for the readers. However, if considered redundant, we are open to either relocating the content to the supplementary material or removing the table entirely.  2. Regarding economic feasibility, both the tests are are inexpensive, widely available, already in use and their combination may offer insight into the inflammatory and thrombotic burden, which is not captured by troponin alone. This is especially relevant for early risk stratification, and possible treatment like, vitamin D supplementation. Moreover, we have added this point to the Discussion subsection clinical implication.  Regarding advantage over troponins and CKMB, our study does not aim to replace these markers but to explore adjunctive biomarkers that may provide additional prognostic or stratification value, particularly in borderline or intermediate-risk presentations.  3. Regarding sample size, we agree that a larger sample size would enhance the strength and generalizability of the findings. However, this study was designed as a preliminary, multicenter exploratory analysis, and the sample size of 300 well exceeded the minimum required sample to detect a moderate effect size based on prior biomarker studies. Having said that, we have also added this to the limitations that larger studies are needed to validate the results before clinical application. |
| Reviewer #5:  This study is of particular interest as it explores the potential of identifying novel biomarkers in the correlation between serum vitamin D levels, fibrinogen, and their ratios, and the severity of acute coronary syndromes. These syndromes include unstable angina, non-ST-elevation myocardial infarction, and ST-elevation myocardial infarction.  The fact that the study was conducted in collaboration with other centers suggests that geographic diversity and generalizability were also taken into consideration.  The hypothesis that the vitamin D/fibrinogen ratio functions as a biomarker is both novel and clinically beneficial. However, given the prevailing circumstances, it is our conviction that the publication system must undergo enhancement to establish a nexus with scientific evidence and clinical applications.  It is imperative that the following points be given due consideration, and that they be linked to the publication.  **Major Points**  1. Overstatement of Conclusions  The vitamin D/fibrinogen ratio demonstrated a slightly stronger negative correlation with ACS severity compared to vitamin D alone (Spearman’s ρ = -0.45 vs. -0.41); however, this difference was not statistically significant (Fisher Z = 0.34, p = 0.73). The manuscript currently presents this ratio as if it is a superior biomarker. Although the numerical difference may appear clinically meaningful, the lack of statistical significance indicates that this claim should be moderated. Given the absence of statistical significance, the authors should emphasize the exploratory nature of this finding and clearly state the need for further validation.  2. Potential Bias Not Adequately Addressed  The logistic regression model does not appear to account for key potential confounding factors that could affect serum vitamin D and fibrinogen levels, such as seasonal variation, sunlight exposure, renal function, vitamin D supplementation, and chronic inflammatory conditions. If this is a multicenter study, addressing potential bias is particularly critical to maintain analytical rigor. The authors should clarify whether these factors were assessed, or explicitly acknowledge their absence as a limitation of the study.  3. Timing of Sample Collection  The timing of serum sample collection in relation to symptom onset or hospital admission is not clearly stated. Considering that fibrinogen is an acute-phase reactant, this timing is critical to properly evaluate its utility as a biomarker. The authors should clarify the timing of sample collection and consider including this aspect in their analysis or discussion.  4. Clinical Interpretation of Vitamin D Levels  While the mean serum vitamin D levels are reported, there is no mention of clinically relevant thresholds (e.g., deficiency <25 nmol/L, insufficiency <50 nmol/L, sufficiency >75 nmol/L) or the number of patients falling into each category. From the perspective of evaluating its utility as a clinical biomarker, the authors are encouraged to analyze and present this information accordingly.  **Minor point**  1. Image Quality of Figure 1  The resolution of Figure 1 is too low to be legible. Please provide a high-resolution version to ensure that readers can accurately interpret the figure.  2. Consistency of Terminology  To avoid reader confusion and improve readability, all abbreviations (e.g., STEMI, NSTEMI, UA, ACS) should be spelled out in full at first mention, followed by the abbreviation in parentheses. Please ensure this is applied consistently throughout the manuscript.  3. Language and Grammar  To enhance the overall readability of the manuscript, please carefully review the English writing and consider the following points:  • Avoidance of Redundant Expressions  Example:  “Vitamin D levels were significantly lower (p < 0.001) and fibrinogen levels significantly higher (p < 0.001)…”  → The word “significantly” is unnecessarily repeated. This can be revised for conciseness.  • Avoidance of Colloquial Expressions  Example:  “To make things simple…”  → This phrasing is too casual for a scientific article. Please replace it with more formal language.  • Correction of Grammatical Errors and Awkward Phrasing  Example:  “…explaining the whole process, including the study benefits to them and the community, the possible risks to them, and the confidentiality and anonymization of their data, to them.”  → The repetition of “to them” makes the sentence unnecessarily redundant.  • Avoidance of Repetitive Word Usage  Example:  “The strength of the correlation (Spearman Rho) showed a significant correlation…”  → The word “correlation” is unnecessarily repeated. Please revise for clarity.  • Simplification of Overly Long and Complex Sentences  In particular, the Methods and Discussion sections contain several overly long and complex sentences, which may reduce readability. Please consider breaking them into shorter, more direct sentences.  4. Reconsideration of Cited Literature  Several of the key references related to vitamin D and cardiovascular disease are over a decade old. Considering the ongoing research in this field, it is recommended to include more recent and high-quality studies, such as randomized controlled trials or meta-analyses (e.g., the VITAL trial). Updating the references will strengthen the scientific relevance of the manuscript. | Thanks for your valuable input into the improvement of the manuscript.  **Major Points:**  1. Regarding overestimation of the conclusion based on the ratio, we appreciate and acknowledge this point and have clearly added this clarification of statistical non-superiority into the various sections of the manuscript. As we have added to the results the statement,  *“However, although the correlation vitamin D/fibrinogen ratio to ACS types was stronger (0.45) as compared to that of vitamin D alone (0.41), it was not significantly stronger statistically (Fisher Z value 0.34, P value 0.73)…”*  Similarly, we have added to the Discussion subsection Vitamin D/Fibrinogen ratio and ACS, as  *“Although this study shows a stronger correlation of vitamin D/fibrinogen ratio to ACS severity, it is not statistically stronger than vitamin D alone with an insignificant Fischer Z value (P = .73).”*  2. Regarding potential bias, we acknowledge your valuable observation. Although vitamin D supplementation, liver or kidney disease, malignancy, anticoagulants were the exclusion criteria mentioned in the methodology, the seasonality and sun-exposure was not addressed, and have been added as one of the limitations, stated as,  *“Another limitation of the study is the lack of information regarding the seasonality, sun-exposure and sub-clinical inflammation that might have affected the results and should be considered while performing future studies in this context.”*  3. Regarding the timing of sample collection, we appreciate your observation. Although previously not mentioned, we have added this to the methodology as all the blood samples are collected at the time of admission, within the first three hours.  4. Regarding clinical interpretation of Vitamin B levels among ACS groups, we really appreciate pointing out this omission. In the revised manuscript, we have added the latest reference of vitamin D levels to the introduction and added the status of vitamin D deficiency among different ACS groups of the cohort.  **Minor point:**  1. Regarding Figure quality, although we have added the prescribed dpi figure, in the revised version we have enhanced the dpi even more.  2. Regarding Consistency of terminology, we have reviewed the manuscript to ensure that all abbreviations (ACS, STEMI, NSTEMI, UA) are spelled out in full at first mention, followed by abbreviation in parentheses and used consistently throughout the text.  3. Regarding Grammer and language, we have revised the manuscript with the help of professional copyediting and proofreading, and all the mentioned error and omissions have been corrected.  4. Regarding citations, we really thank the suggestion to strengthen the manuscript by incorporating more recent and high-quality studies and we have now included the VITAL trial and its continuation which is a landmark randomized controlled trial evaluating the role of vitamin D supplementation in cardiovascular outcomes.  At the same time, we respectfully emphasize that several references that are more than a decade old were deliberately retained because they provide foundational insights into the biological mechanisms, epidemiological associations, and historical development of vitamin D and fibrinogen research in cardiovascular disease. These include seminal studies such as:  *Pilz et al. (2008) – A cornerstone observational study linking low vitamin D to cardiovascular mortality.  *Wang et al. (2008) – A key meta-analysis establishing the association between vitamin D status and incident cardiovascular events.  *Danesh et al. (2005) – A major pooled analysis on fibrinogen and vascular risk, still widely cited in biomarker literature.  Out of 28 citations only 7 are older than 2012, while most of them are older than 2015 i.e; upto 75%. The older references continue to be cited in recent systematic reviews and clinical guidelines due to their methodological robustness and ongoing relevance.  Therefore, while we have updated the manuscript with newer studies, we have retained select older references to maintain a comprehensive and historically contextualized discussion. All retained references were selected for their scientific relevance, not publication age. |
| Reviewer #6:  Point 1: Lack of systematic approach: As stated in the study, the vitamin D/fibrinogen ratio appears an ad hoc metric. Strengthen the rationale by citing pathophysiology. Suggestion: Please outline how low vitamin D contributes to endothelial dysfunction and high fibrinogen to thrombosis in ACS, then explain why their ratio would reflect net thrombo‐protective status.  Point 2: Power calculation mismatch: The methods claim a required sample size of 60 (4% population prevalence), yet you enrolled 300 six times that number?? Suggestion : Recalculate the power analysis using effect sizes from prior studies of vitamin D and fibrinogen in ACS to demonstrate that n = 300 yields >80% power to detect clinically meaningful differences across three groups.  Point 3: This study has multiple pairwise comparisons (UA vs. NSTEMI vs. STEMI) without adjustment. Suggestion: Try to apply a correction (Bonferroni or Holm) to control familywise error, and report both raw and adjusted p-values." | Point 1: We really appreciate this important point. While the rationale for using the vitamin D/fibrinogen ratio was discussed, we agree it can be strengthened by elaborating on the pathophysiological mechanisms linking both components to ACS. In this context we have added a paragraph to the introduction section elaborating the rationale further.  Point 2: The power analysis, using G*Power version 3.1 has been done, showing power of more than 97% for sample size of 300, showing clinically meaningful difference among the three ACS groups.  Point 3: Thanks for this valuable suggestion, and we have applied Bonferroni and Games-Howell as stated in the results as,  *“Post hoc pairwise comparisons with Bonferroni and Games-Howell adjustments confirmed statistically significant differences in the vitamin D/fibrinogen ratio between all ACS subtypes (UA, NSTEMI, STEMI; all p < 0.01). The ratio showed a stepwise decline from UA to STEMI, supporting its association with ACS severity. Similarly, serum vitamin D levels were significantly lower in STEMI compared to NSTEMI and UA (p < 0.001). Fibrinogen levels were significantly higher in NSTEMI and STEMI compared to UA (p < 0.001), though the difference between NSTEMI and STEMI was not statistically significant (p = 1.000, Bonferroni) and 0.89 (Games-Howell)”.* |
