# Supplementary material for: Relationship of vitamin D, fibrinogen and their ratio with acute coronary syndrome: A comparative analysis of unstable angina, NSTEMI, and STEMI
Source: PLoS One. 2025 Sep 5;20(9):e0331853. doi: 10.1371/journal.pone.0331853 (PMC12412923; doi:10.1371/journal.pone.0331853)
Supplement: Attachment — Submitted filename: Response to the reviewers 2nd revision.docx [file pone.0331853.s004.docx]

Response to the reviewers

| Reviewers’ comments | Response |
| --- | --- |
| Reviewer #1:  The manuscript "Relationship of Vitamin D, Fibrinogen and Their Ratio with Acute Coronary Syndrome" has made substantial improvements since the prior review, particularly in addressing clinical implications, statistical power, and manuscript clarity. However, several key issues require further attention to ensure compliance with PLOS ONE standards and enhance the rigor of the study. 1. Data Availability and Transparency：The current data availability statement is insufficient and vague, failing to meet PLOS ONE’s requirement for unrestricted data sharing. 2. Statistical Methods and Reporting： While multiple comparisons were corrected using Bonferroni and Games-Howell tests, the rationale for choosing these methods and their impact on results is unclear. Additionally, potential collinearity between vitamin D and the vitamin D/fibrinogen ratio was not assessed. 3. Research Design Limitations：The non-probability convenience sampling introduces selection bias, but the discussion lacks sensitivity analysis or sub-group comparisons to assess its impact. 4. Mechanistic and Clinical Context：The discussion of biological plausibility for the vitamin D/fibrinogen ratio remains superficial, and clinical application suggestions are not contextualized with practical challenges. 5. Language and Formatting: Minor grammatical errors, inconsistent terminology, and formatting issues persist, affecting readability. 6. Ethical and Compliance Details: While ethics approval is mentioned, the methods lack detail on patient consent processes and data anonymization measures.  The study provides valuable insights into novel biomarkers for ACS, but further refinements are necessary to address data transparency, statistical rigor, and clinical relevance. Once the above issues are resolved, the manuscript will be suitable for publication in PLOS ONE. | We really appreciate valuable comments towards the improvement of the manuscript. We are thankful to your kind words and appreciation of the authors’ hard work.  1. Regarding data availability and transparency, we have added a statement regarding the data availability according to PlosOne guidelines and attached a supplementary file.  2. Regarding point 2, we would clarify that we have mentioned the rationale behind using Bonferroni and Games-Howell post-hoc tests is the Statistical Analysis section, stating;  *“Analysis of variance (ANOVA) followed by the Kruskal-Wallis test was performed to show the difference among the ACS groups. The Games-Howell post hoc was performed to evaluate the inter-group pairwise differences in Vitamin D, Fibrinogen levels, and the VitD/Fibrinogen ratio (Unstable angina VS NSTEMI VS STEMI)”.*  However, to improve upon this, we have revised and rewrote it as,  “*Bonferroni post-hoc tests were used for pairwise comparisons where homogeneity of variance was assumed (as tested using Levene’s test), while Games-Howell was employed where variances were unequal. This approach ensures rigorous control over Type I error in both parametric and non-parametric contexts*.”  Regarding collinearity, we performed collinearity diagnostics using Variance Inflation Factor (VIF) and the statistics are added to the results as, *“Collinearity diagnostics using Variance Inflation Factor (VIF) showed values of 3.04, 1.56, and 1.03 for vitamin D and vitamin D/fibrinogen ratio, fibrinogen and vitamin D/fibrinogen ratio, and vitamin D to fibrinogen, respectively. All these values are less than 5, indicating no significant multicollinearity (p < .05 for all)”.*  3. Regarding sensitivity analysis, we have performed the sensitivity analysis in the revised version and mentioned in the results as,  *“To further mitigate the risk of selection bias, stratified sensitivity analyses across the three study sites were also performed, which showed consistent trends in biomarker levels and their associations with ACS severity across the three countries”.*  4. Regarding the mechanistic, the discussion on the biological plausibility of vitamin D/fibrinogen ratio has been discussed in details in the introduction section, by first discussing the roles of vitamin D and fibrinogen separately, then discussing their contrasting effects and then stating, *“Although the effects of vitamin D and fibrinogen on vascular health and coronary arteries have been studied individually, their combined effect has never been studied previously [15]. These contrasting effects of vitamin D and serum fibrinogen may give rise to the hypothesis that the interplay of these two important biomarkers could affect the course and severity of CAD. Vitamin D deficiency and high serum fibrinogen levels may play a synergistic role in coronary vascular inflammation, endothelial dysfunction, platelet aggregation and atherosclerosis, thus adversely affecting the ACS severity.”*  Restating all these will not only lengthen the discussion but also cause redundancy. However, we have further explained the clinical applicability and practical challenges of the ratio in the end of clinical implications subsection as,  *“Clinically, however, implementation challenges include assay availability, inter-laboratory variation, and lack of standardized cutoffs. These barriers must be addressed before routine use in ACS triage.”*  5. Regarding minor language and formatting issues, we are really thankful to point this drawback out. We have used a professional proofreader and copy-editor has been used to improve the revised manuscript.  6. Regarding detail on patient consent processes and data anonymization measures, it has already been mentioned in the Material and methods section as,  “*A written informed consent was taken from each patient explaining the study process, including the potential benefits to participants and the community, possible risks, and measures to ensure confidentiality and data anonymization.”*  Once again, thank you for appreciating our efforts and providing valuable insights and input in the improvement of the manuscript. |
| Reviewer #2: I am very pleased to see this revised manuscript. The quality of this manuscript has significantly improved, and the readability of the article has also significantly improved. The conclusion of the article is also consistent with the overall situation of this clinical study. But there are still some issues that we hope to improve before publication. Firstly, the title of the discussion section lacks numbering. A reliable title can further enhance the readability of the overall article structure and clearly display the structure of the article. If you do not intend to change the font size of the main title and subtitle, it is recommended to add numbering. Secondly, discuss the structure of the article in the discussion section. The discussion section does not simply list the sources of inspiration for this article. I hope to see further thinking and meaningful discussions. And why is content similar to a conclusion placed at the beginning of the discussion? | We are really thankful for your encouragement and valuable comments.  Regarding numbering and font size, we totally agree with your valuable suggestion. Since the font size is in accordance with the journal’s suggested template, we have added numbering to the Discussion sections. This will enhance the clarity and navigation.  Regarding suggestion for a meaningful discussion, we really appreciate your insightful analysis. We have revised the discussion and added deeper mechanistic of vitamin D, fibrinogen and their ratio into the respective subsections in the discussion, keeping in view the word limits and redundancy. We hope that this will add to the meaningful discussion.  Regarding the opening paragraph of the discussion, we agree that it is resembling conclusion and we have restructured it according and now discussion starts with the key findings of this study in context with previous literature on the pathophysiologic processes of vitamin D and fibrinogen in CAD.  We are really thankful for your constructive suggestions. We believe these changes have substantially improved the clarity, structure, and depth of the discussion. |
| Reviewer #5: General comments: This revised manuscript shows significant improvements in clarity, structure, and scientific rigor. The authors have thoroughly and clearly addressed all of the previous reviewers' comments. The study design is observational and based on convenience sampling, but it is appropriately justified in the context of a clinical setting and multi-center logistics.  Minor suggestions for improvement: • Please confirm the consistency of terminology, particularly the standardized notation for the “vitamin D/fibrinogen ratio.” • Figure 1 should be more appropriately integrated into the flow of the narrative in the results section. • The authors should concisely emphasize that while the vitamin D/fibrinogen ratio is not statistically superior, its clinical utility as a composite marker is a promising area. | We are really thankful to you for the encouraging feedback and acknowledging the revisions made to improve the manuscript’s clarity, structure, and methodological transparency. We are grateful for the constructive suggestions and have addressed them in the revised version one by one.  Regarding consistency of the terminology, we have reviewed and confirmed that uniform terminology is used throughout the manuscript. Any alternative phrases have been replaced by uniform standard phrasing, for example vitamin D/fibrinogen ratio.  Regarding the narration of Figure 1 in the results section, we agree with your suggestion and added the narration before the figure as,  “*Figure 1 illustrates the distribution of vitamin D, fibrinogen, and their ratio across ACS categories. A descending trend in vitamin D and the vitamin D/fibrinogen ratio, and an ascending trend in fibrinogen levels, are clearly evident from unstable angina to STEMI, reinforcing the statistical findings from the ANOVA and post-hoc comparisons.”*  Regarding the emphasis on the statistical non-superiority of the ratio over vitamin D alone, we totally agree to your suggestion and an emphasis is made on this in the discussion section, adding,  “*Although this superiority is not statistically significant to vitamin D alone, its dual representation of anti-inflammatory and prothrombotic axes may enhance clinical relevance as a composite risk marker in ACS assessment”.*  We believe these refinements have further improved the clarity, scientific integrity, and clinical value of our work. |
|  |  |
|  |  |
