# Supplementary material for: Relationship of vitamin D, fibrinogen and their ratio with acute coronary syndrome: A comparative analysis of unstable angina, NSTEMI, and STEMI
Source: PLoS One. 2025 Sep 5;20(9):e0331853. doi: 10.1371/journal.pone.0331853 (PMC12412923; doi:10.1371/journal.pone.0331853)
Supplement: Attachment — Submitted filename: Point by Point Response 3rd.docx [file pone.0331853.s005.docx]

Point by Point Response 3^rd^

| Reviewer Comment | Response |
| --- | --- |
| Reviewer #5: The authors partially discuss the clinical significance of the vitamin D/fibrinogen ratio. As pointed out in the previous section, I believe that the practical significance of utilizing these findings is the core of this paper, so it is necessary to state this clearly. I believe that adding further detailed explanations will enhance the clinical application value of the research results.  Minor comments Clinical Value of the Ratio Needs Further Elaboration The ratio showed a stronger correlation with ACS severity than vitamin D alone, though the difference was not statistically significant. Authors should better clarify the potential clinical utility of this ratio — e.g., how it could complement existing biomarkers like troponin or CRP, and the practicality of dual-assay implementation in triage. | We are really thankful to your valuable comments to improve the quality of the manuscript.  Regarding further elaborating the practical significance and clinical application value of the vitamin D/fibrinogen ratio, we have further expanded the clinical implications subsection of the Discussion that while the vitamin D/fibrinogen ratio did not show a statistically significant superiority over vitamin D alone in our cohort, it demonstrated a consistent trend toward stronger correlation with ACS severity and remained an independent predictor after multivariate analysis. We further stated that the ratio integrates two biologically opposing processes, i.e., anti-inflammatory/antithrombotic effects of vitamin D and prothrombotic/pro-inflammatory effects of fibrinogen, into a single index, potentially providing a more holistic measure of thrombo-inflammatory status in ACS.  Minor Comments:  Regarding complementing the established biomarkers, we have added a specific discussion on how the ratio could be incorporated into the current biomarker framework, like troponins or CRP.  Regarding the practicality of Implementation, we have stated that both vitamin D and fibrinogen assays are inexpensive, widely available, and already used in clinical laboratories; hence, in acute triage, this ratio could assist in early identification of higher-risk patients in whom the troponins and CRP are equivocal. |
